# Establishment of a High-risk MDS/AML Cell Line YCU-AML1 and its Xenograft Model Harboring t(3;3) and Monosomy 7

**DOI:** 10.1097/HS9.0000000000000469

**Published:** 2020-09-17

**Authors:** Hiroyoshi Kunimoto, Yumi Fukuchi, Koichi Murakami, Junji Ikeda, Hiroshi Teranaka, Ikuma Kato, Takuya Miyazaki, Makiko Enaka, Takayuki Mitsuhashi, Etsuko Yamazaki, Kaori Kameyama, Mitsuru Murata, Shinichiro Okamoto, Hideaki Nakajima

**Affiliations:** 1 Department of Stem Cell and Immune Regulation, Yokohama City University Graduate School of Medicine, Kanazawa-ku, Yokohama, Japan; 2 Department of Pathophysiology, Hoshi University School of Pharmacy and Pharmaceutical Sciences, Shinagawa-ku, Tokyo, Japan; 3 Department of Pediatrics, Yokohama City University Graduate School of Medicine, Kanazawa-ku, Yokohama, Japan; 4 Department of Molecular Pathology, Yokohama City University Graduate School of Medicine, Kanazawa-ku, Yokohama, Japan; 5 Department of Laboratory Medicine, Keio University School of Medicine, Shinjuku-ku, Tokyo, Japan; 6 Clinical Laboratory Department, Yokohama City University Hospital, Kanazawa-ku, Yokohama, Japan; 7 Department of Pathology, Keio University School of Medicine, Shinjuku-ku, Tokyo, Japan; 8 Division of Hematology, Department of Medicine, Keio University School of Medicine, Shinjuku-ku, Tokyo, Japan.

## Abstract

Acute myeloid leukemia (AML) or myelodysplastic syndromes (MDS) with both inv(3)(q21q26.2)/t(3;3)(q21;q26.2) and monosomy 7 defines an extremely aggressive myeloid cancer whose molecular pathogenesis and optimal therapeutic strategy still remain unclear. We established a new MDS/AML cell line, YCU-AML1, and its patient-derived xenograft (PDX) model from a high-risk MDS patient who later transformed into AML harboring both t(3;3)(q21;q26.2) and monosomy 7. YCU-AML1 cells propagated in co-culture system with stromal cells in granulocyte macrophage colony-stimulating factor (GM-CSF)-dependent manner. CD34^+^ bone marrow cells derived from our PDX model showed high *EVI1* and low *GATA2* expression. Moreover, mutational profile of our MDS/AML model was consistent with recently published mutational spectrum of myeloid malignancies with inv(3)/t(3;3). These data suggest that YCU-AML1 cells and its MDS/AML model strongly mimics a high-risk human myeloid cancer with inv(3)(q21q26.2)/t(3;3)(q21;q26.2) and monosomy 7 in terms of both clinical phenotype and molecular basis. We believe our model can be used as a feasible tool to further explore molecular pathogenesis and novel treatment strategy of high-risk MDS/AML with t(3;3)(q21;q26.2) and monosomy 7.

## Introduction

Acute myeloid leukemia (AML) is a myeloid malignancy characterized by differentiation block of myeloid cells and relentless proliferation of immature myeloid blasts. Among AML, acute myeloid leukemia with myelodysplasia-related changes (AML-MRC) comprises a unique category of AML with morphological features of myelodysplasia, a prior history of myelodysplastic syndromes (MDS) or myelodysplastic/myeloproliferative neoplasms (MDS/MPN), or MDS-related cytogenetic abnormalities.^1^ Previous studies have shown that AML-MRC has a significantly worse prognosis and a lower rate of complete remission than in other AML subtypes,^1,2^ making it a challenging clinical entity. Among various clinical parameters, cytogenetics remains to be the most intense prognostic indicator in both AML and MDS. AML or MDS with inv(3)(q21q26.2) or t(3;3)(q21;q26.2) defines an aggressive myeloid cancer with short survival.^3,4^ More than half of cases with inv(3)(q21q26.2) or t(3;3)(q21;q26.2) harbor additional monosomy 7 and these patients are associated with an even worse prognosis.^3,4^ Although recent genomics studies have uncovered mutational landscape, dynamics of clonal evolution and their prognostic relevance of AML and MDS,^5–7^ these studies also revealed complexity and heterogeneity of genetic background of these diseases. Hence their exact molecular pathogenesis, including that of AML-MRC, still remains elusive.

In an attempt to elucidate molecular basis of these myeloid cancers, a number of patient-derived xenografts (PDXs) of de novo AML have been developed.^8^ The original patient donors of these models include AML with recurrent genetic abnormalities, such as AML with inv(16)^9,10^ or AML with *BCR-ABL1*.^11^ On the other hand, establishing PDXs of MDS or AML-MRC has been a challenge unless primary patient bone marrow (BM) cells are injected directly into the murine BM cavity with patient-derived mesenchymal stromal cells.^12^ Although a stroma-dependent AML cell line with inv(3) and monosomy 7, OCI-AML-20, has been established,^13^ highly penetrant in vivo PDX AML-MRC model harboring both inv(3)(q21q26.2)/t(3;3)(q21;q26.2) and monosomy 7 has yet to be reported. Here, we established a new AML-MRC cell line, YCU-AML1, together with an in vivo PDX model of human AML-MRC which mimics the original case harboring both t(3;3)(q21;q26.2) and monosomy 7, two major high-risk cytogenetic abnormalities which frequently co-occur in high-risk AML and MDS. This model can be easily established with high penetrance by simply injecting primary patient BM cells into tail vein of irradiated NOD/SCID-IL2Rγ^null^ (NSG) mice, which is reproducible by serial transplantation. YCU-AML1 cells propagated in vitro in co-culture system with stromal cells and showed hypersensitivity to granulocyte macrophage colony-stimulating factor (GM-CSF). Quantitative reverse transcriptase-polymerase chain reaction (RT-PCR) and targeted deep sequencing of YCU-AML1 cells confirmed high *EVI1* expression and mutational profile consistent with recently published mutational spectrum of myeloid malignancies with inv(3)/t(3;3).^14^ Therefore, YCU-AML1 cells and our PDX model can be used as functional tools for seeking molecular basis of high-risk human AML-MRC with t(3;3)(q21;q26.2) and monosomy 7.

## Results

### Clinical characteristics of the original MDS/AML patient

**Figure 1:**
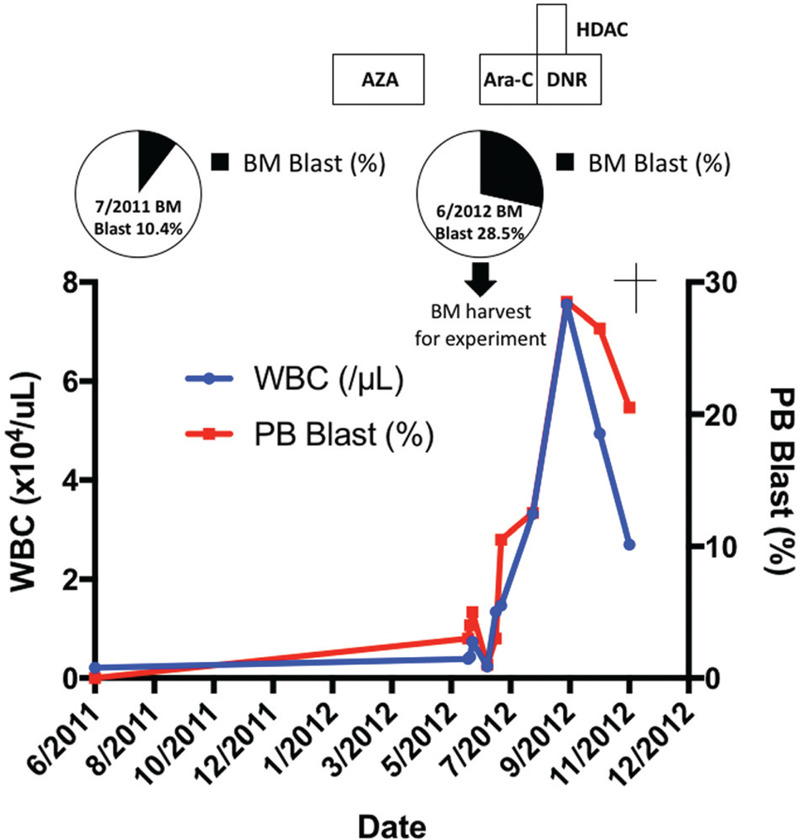
**Clinical course of the patient.** The lower line graph shows white blood cell counts (WBC, blue line) and percentages of blasts in peripheral blood (PB Blasts, red line) at the indicated time points. The middle pie charts represent percentages of blasts in bone marrow at the indicated time points (black portion). Patient BM sample was collected at the time of leukemic transformation (second BM aspiration) as depicted in the figure. The upper rectangles depict chemotherapeutic regimens performed at the indicated time points. AZA = Azacytidine. Ara-C = Cytarabine, DNR = Daunorubicine, HDAC = High-dose cytarabine.

**Figure 2:**
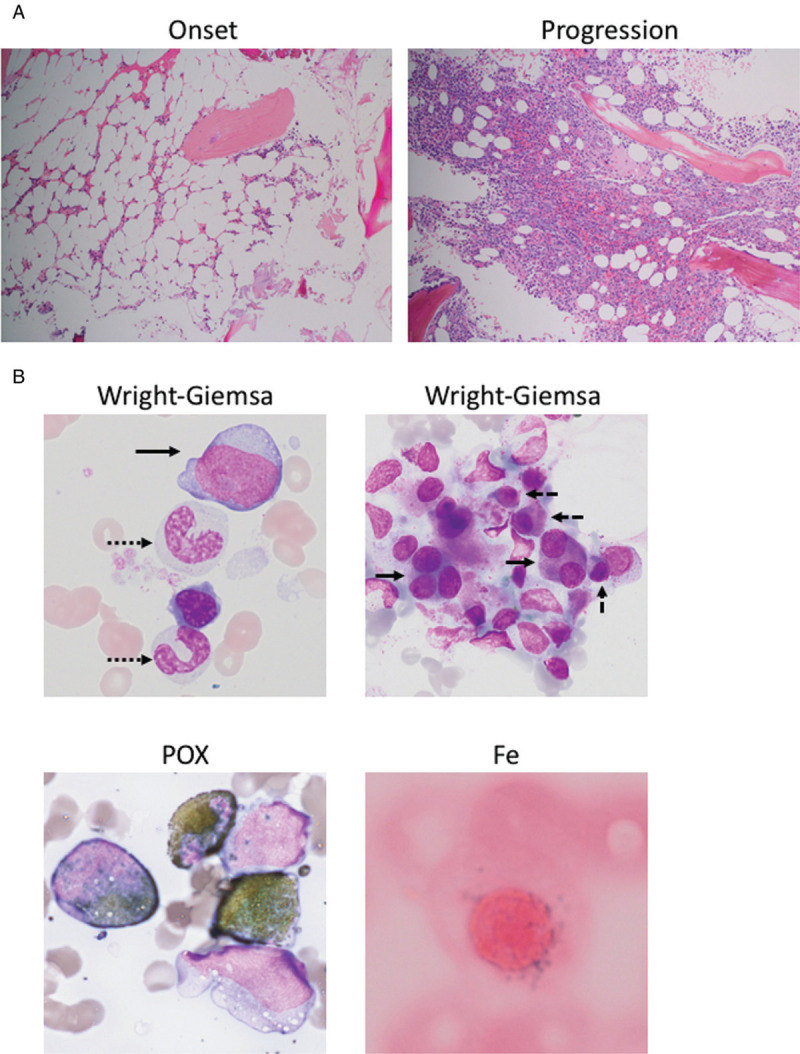
**Bone marrow pathology and dysplasia.** A. Hematoxylin and eosin staining of bone marrow biopsy specimens at the time of disease onset (left panel) and progression to leukemia (right panel). Bone marrow was hypocellular at the time of disease onset whereas it became hypercellular at the time of leukemic transformation. B. Wright-Giemsa staining (upper panels), POX staining (lower left) and iron staining (lower right) of bone marrow smears. Wright-Giemsa staining shows a myeloblast with vacuoles (upper left panel, solid arrow), granulocytes with decreased granules (upper left panel, dashed arrow), a bilobated megakaryocyte, megakaryocyte with multinucleation/nuclear hypolobation (upper right panel, solid arrow) and micromegakaryocytes (upper right panel, dashed arrow). POX staining shows POX(+) and POX(−) myeloblasts. Iron staining shows a ring sideroblast.

Our MDS/AML PDX model was originally established from a 62-year old male patient diagnosed with AML-MRC (MDS/AML). The patient's clinical course, including WBC counts, PB/BM blast percentages and therapeutic regimens, is shown in Figure 1. Before diagnosis, the patient had a history of type 2 diabetes mellitus and ulcerative colitis, but no history of cancer or previous treatment with chemotherapy/radiation. Initially, the patient showed low WBC count and macrocytic anemia and BM exam revealed hypocellularity (Fig. 2A), dysgranulopoiesis (decreased granules) and blast percentage of 10.4%, leading to the diagnosis of MDS-EB2. The patient's anemia exacerbated and he gradually became transfusion-dependent, which encouraged his attending to start treatment with Azacytidine (AZA). Despite 3 courses of AZA treatment, the patient showed increased PB blast percentage and the second BM exam demonstrated hypercellularity, increased myeloperoxidase (POX) positive and negative blasts with vacuoles, dysgranulopoiesis (decreased granules), dysmegakaryopoiesis (micromegakaryocytes, nuclear hypolobation, multinucleation), dyserythropoiesis (ring sideroblasts) and blast percentage of 28.5%, consistent with leukemic transformation (Fig. 1 and 2A and B). Although the initial BM exam showed normal karyotype, the second BM exam revealed acquisition of t(3;3)(q21;q26.2) and monosomy 7 in 19 out of 20 analyzed cells in metaphases (Table 1). Flow cytometric analysis confirmed high positivity of HLA-DR, CD13, CD33, CD34, and CD38 (Table 2). Aberrant CD7 expression was also observed as previously reported in AML with inv(3)(q21q26.2)/t(3;3)(q21;q26.2) (Table 2).^16^ BM mononuclear cells (BMMNCs) were isolated from the second BM sample and cryopreserved. Treatment with intravenous cytarabine (Ara-C) temporary reduced PB blasts. However, the patient soon became resistant to Ara-C and therefore the attendings started induction chemotherapy with high-dose Ara-C (HDAC) and daunorubicin (DNR). Although the induction therapy was effective in reducing PB blasts, the patient suffered from multiple episodes of blood stream infections which precluded further treatment with chemotherapy. The patient developed multiple organ failure and died 5 months after leukemic transformation (Fig. 1).

**Table 1:**
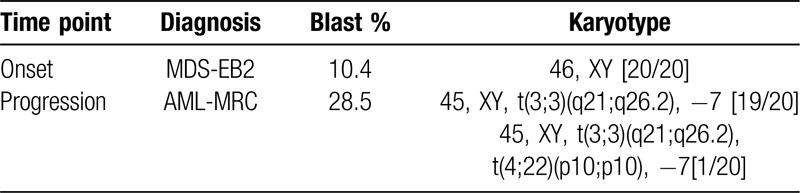
Patient karyotype.

**Table 2:**
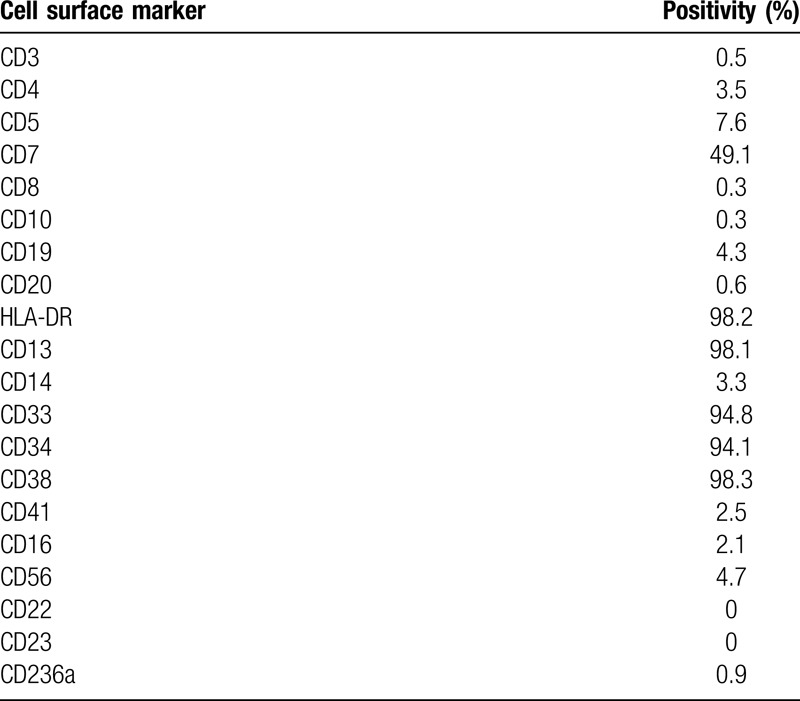
Flow cytometric analysis of the BM.

### Establishment of YCU-AML1 and its PDX model

**Figure 3:**
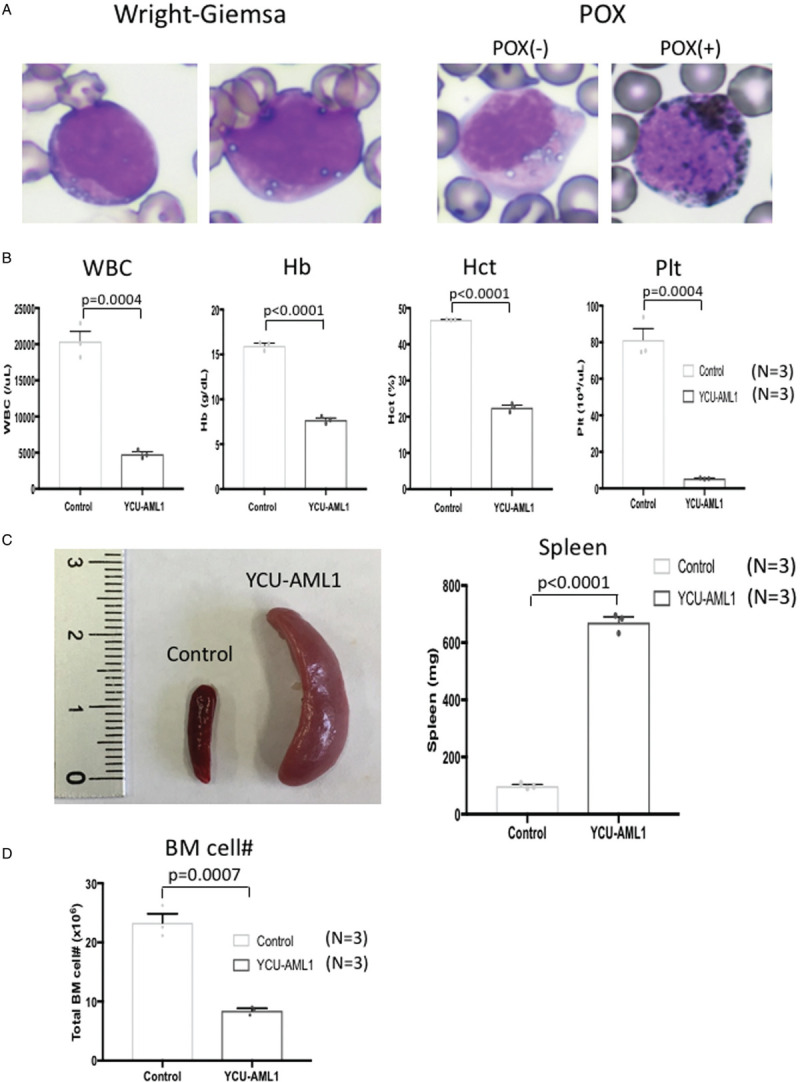
**Development of MDS/AML in YCU-AML1 PDX model.** A. Wright-Giemsa staining (left panels) and POX staining (right panels) of peripheral blood smears derived from primary YCU-AML1 mice. Wright-Giemsa staining shows myeloblasts with vacuoles and POX staining shows POX(+) and POX(−) myeloblasts, which are similar to the original case. B. Complete blood counts of primary YCU-AML1 mice and age-matched controls at 8 weeks from transplantation (N = 3 for each group). C. Representative image of spleens (left) and bar graph of spleen weights from each group (right) at 8 weeks from transplantation (N = 3 for each group). D. The number of whole bone marrow cells derived from bilateral femurs, tibias and iliac bones from each group at 8 weeks from transplantation (N = 3 for each group). 1.0 × 10^6^ patient BMMNCs per mouse were injected. An unpaired Student's *t* test was used for p values. Data shown in graphs indicate mean ± S.E.M.

In order to further explore molecular pathogenesis of MDS/AML with t(3;3)(q21;q26.2) and monosomy 7, we sought to establish in vitro culture system as well as in vivo PDX model from the original patient sample. Tail vein injection of patient BMMNCs, which were harvested at leukemic transformation, to irradiated NSG mice led to engraftment and development of lethal leukemia within 2 months in all injected mice (injected 1.0 × 106 cells per mouse). Cryopreserved BM cells from the xenograft continuously propagated in co-culture system with OP9 stromal cells with GM-CSF supplementation for more than 3 months. Thus YCU-AML1 cells, a new MDS/AML cell line, and its primary PDX model (primary YCU-AML1 mice) were established. Wright-Giemsa and POX staining of the PB smear from primary YCU-AML1 mice 8 weeks after transplant showed both POX positive and negative blasts with vacuoles similar to those seen in the original case (Fig. 3A). Complete blood counts revealed significant leukopenia (mean 4800/μL, p = 0.0004; control 20,400/μL), anemia (mean Hb 7.7 g/dL, p < 0.0001; control 15.97 g/dL, mean Hct 22.53%, p < 0.0001; control 46.8%) and thrombocytopenia (mean 5.43 × 10^4^/μL, p = 0.0004; control 81.3 × 10^4^/μL) in primary YCU-AML1 mice compared to controls (Fig. 3B).

**Figure 4:**
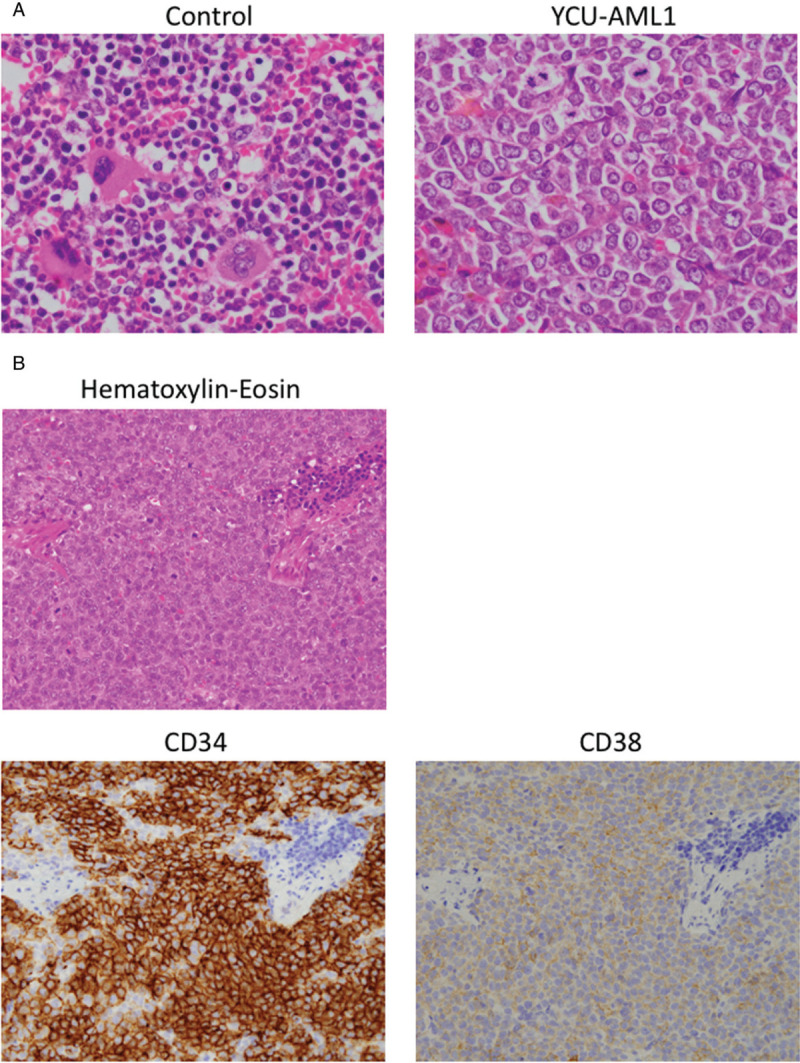
**Pathological evidence of leukemia development in YCU-AML1 mice.** A. Hematoxylin and eosin staining of vertebra specimens derived from control (left) and primary YCU-AML1 mice (right) at 8 weeks from transplantation. B. Hematoxylin-Eosin staining (upper panel) and immunohistochemistry for CD34 (lower left) and CD38 (lower right) of a spleen derived from primary YCU-AML1 mice at 8 weeks from transplantation.

To further clarify leukemia progression, we examined BM and spleen of primary YCU-AML1 mice. Although these mice demonstrated significantly lower BMMNC count compared to controls, primary YCU-AML1 mice developed marked splenomegaly (mean 671 mg, p < 0.0001; control 98.67 mg) (Fig. 3C and D). Moreover, pathological examination of the BM and spleen revealed robust infiltration of blasts and significant destruction of normal spleen architecture (Fig. 4A and B). Importantly, immunohistochemical examination confirmed infiltration of CD34^+^/CD38^+^ blasts in the spleen, consistent with aggressive leukemia development in vivo (Fig. 4B). These data suggest that tail vein injection of patient BMMNCs to irradiated NSG mice led to the development of aggressive leukemia in vivo.

**Figure 5:**
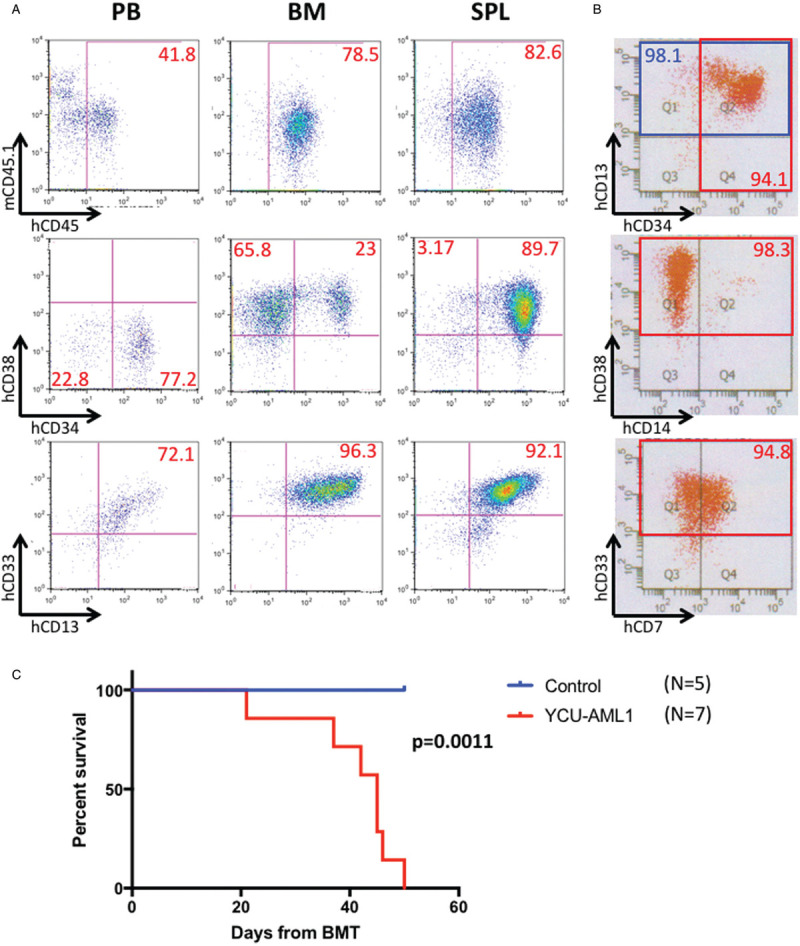
**Leukemic immunophenotype and survival of YCU-AML1 mice.** A. Representative immunophenotype of peripheral blood (PB), bone marrow (BM) and spleen (SPL) derived from primary YCU-AML1 mice at 8 weeks from transplantation, with the percentage of each fraction indicated. Cells presented for hCD34/hCD38 and hCD13/hCD33 are gated on hCD45^+^ cells. B. Representative immunophenotype of the original patient BM cells injected to primary NSG mice. C. Kaplan-Meier survival curve of age-matched control (N = 5) and primary YCU-AML1 mice (N = 7).

We next sought to characterize immunophenotype of the blast cells. Moribund primary YCU-AML1 mice around 8 weeks after transplant showed increased CD34^+^/CD38^−^ and CD13^+^/CD33^+^ blast cells in the PB (Fig. 5A). Interestingly, the immunophenotype for CD34 and CD38 was slightly different in the BM and spleen. Two subpopulations (CD34^+^/CD38^+^ and CD34^−^/CD38^+^) were observed in the BM, whereas CD34^+^/CD38^+^ fraction was the only major population in the spleen (Fig. 5A). These cells were mostly CD13^+^/CD33^+^ in both lesions. Of note, the original patient BM cells injected to primary NSG mice exhibited similar immunophenotype, including high CD34, CD38, CD13 and CD33 positivity (Fig. 5B). These data indicate that expanded blasts in our PDX model mimic the immunophenotypic features of the original case. However, variety in CD34 positivity between the BM and spleen may reflect functional difference as a leukemic microenvironment; BM of the NSG mice supports engraftment of two subpopulations (CD34^+^/CD38^+^ and CD34^−^/CD38^+^) whereas the spleen only supports the expansion of CD34^+^/CD38^+^ fraction. Survival analysis revealed that primary YCU-AML1 mice had significantly impaired survival compared with age-matched controls (median survival 45 days vs > 50 days, p = 0.0011), consistent with lethal leukemia development in vivo (Fig. 5C).

**Figure 6:**
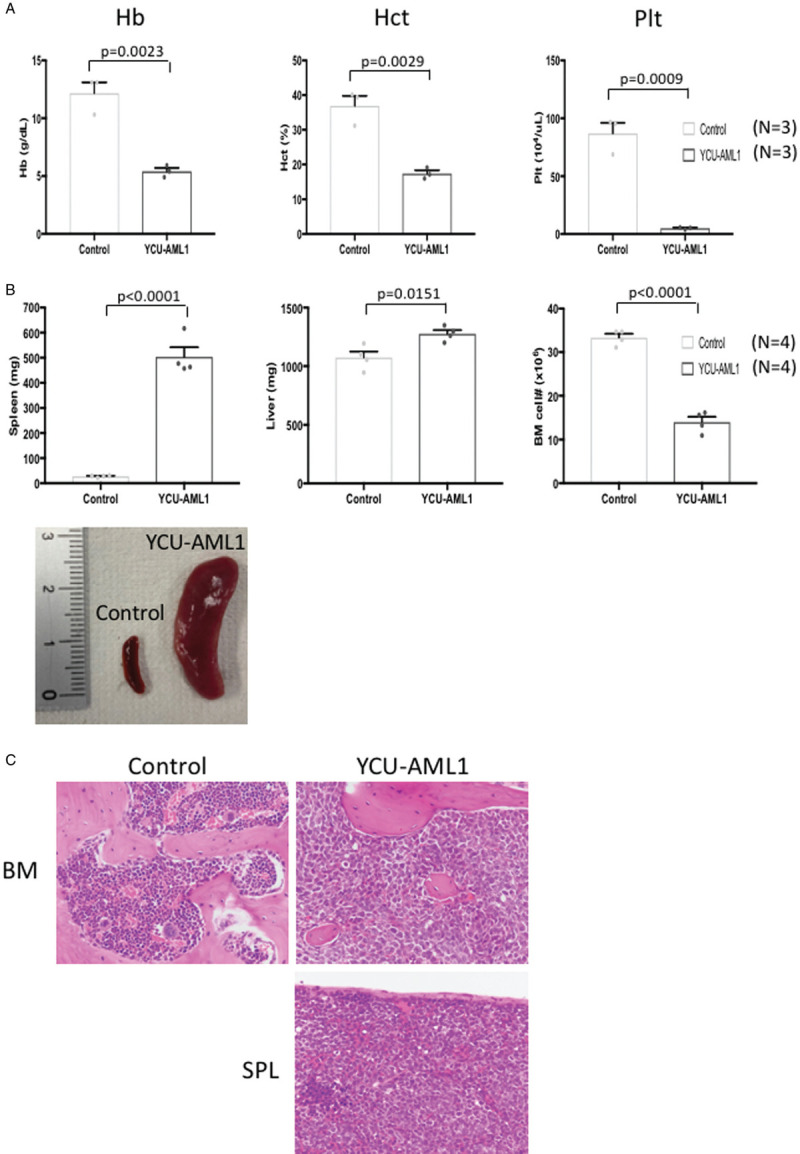
**Disease propagation in secondary YCU-AML1 mice.** A. Hemoglobin (Hb), hematocrit (Hct) and platelet counts of secondary YCU-AML1 mice and age-matched controls at 8 weeks from transplantation (N = 3 for each group). B. Representative image of spleens (lower left) and bar graphs of spleen weights, liver weights and the number of whole BM cells derived from bilateral femurs, tibias and iliac bones from each group at 8 weeks from transplantation (N = 4 for each group). C. Hematoxylin and eosin staining of vertebra (upper panel) and spleen (lower right) specimens derived from control (left) and secondary YCU-AML1 mice (right) at 8 weeks from transplantation.

**Figure 7:**
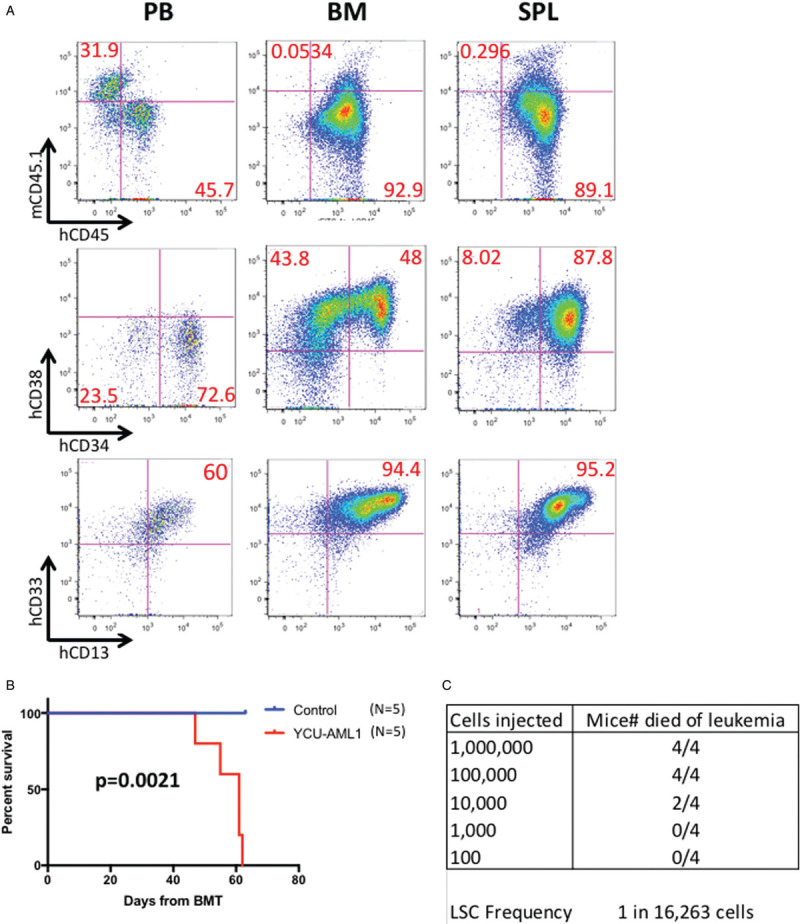
**Reproduced moribund leukemia in secondary YCU-AML1 mice and LSC frequency.** A. Representative immunophenotype of PB, BM, and SPL derived from secondary YCU-AML1 mice at 8 weeks from transplantation, with the percentage of each fraction indicated. Cells presented for hCD34/hCD38 and hCD13/hCD33 are gated on hCD45^+^ cells. B. Kaplan-Meier survival curve of age-matched control (N = 5) and secondary YCU-AML1 mice (N = 5). C. LSC frequency calculated by limiting dilution assay and regression analysis using L-Calc software.

In order to seek if our PDX model is reproducible in vivo, we serially transplanted cryopreserved BM cells derived from primary YCU-AML1 mice to irradiated NSG mice (secondary YCU-AML1 mice). As expected, secondary YCU-AML1 mice also showed significant anemia, thrombocytopenia, organomegaly and decreased BMMNC count as compared to controls (Fig. 6A and B). Pathological examination demonstrated massive infiltration of blasts in the BM and spleen (Fig. 6C). In addition, flow cytometric analysis confirmed similar expansion of the 2 subpopulations (CD34^+^/CD38^+^ and CD34^−^/CD38^+^) and CD13^+^/CD33^+^ blast cells in the BM and spleen (Fig. 7A). Most importantly, all secondary recipients succumbed to leukemia progression within 2 months from transplant. Overall, these data suggest that YCU-AML1 cells can propagate and reproduce similar myeloid leukemia in vivo. Notably, limiting dilution assay and regression analysis using L-Calc software uncovered leukemic stem cell (LSC) frequency as 1 in 16,263 cells in YCU-AML1 mice (Fig. 7C).

**Figure 8:**
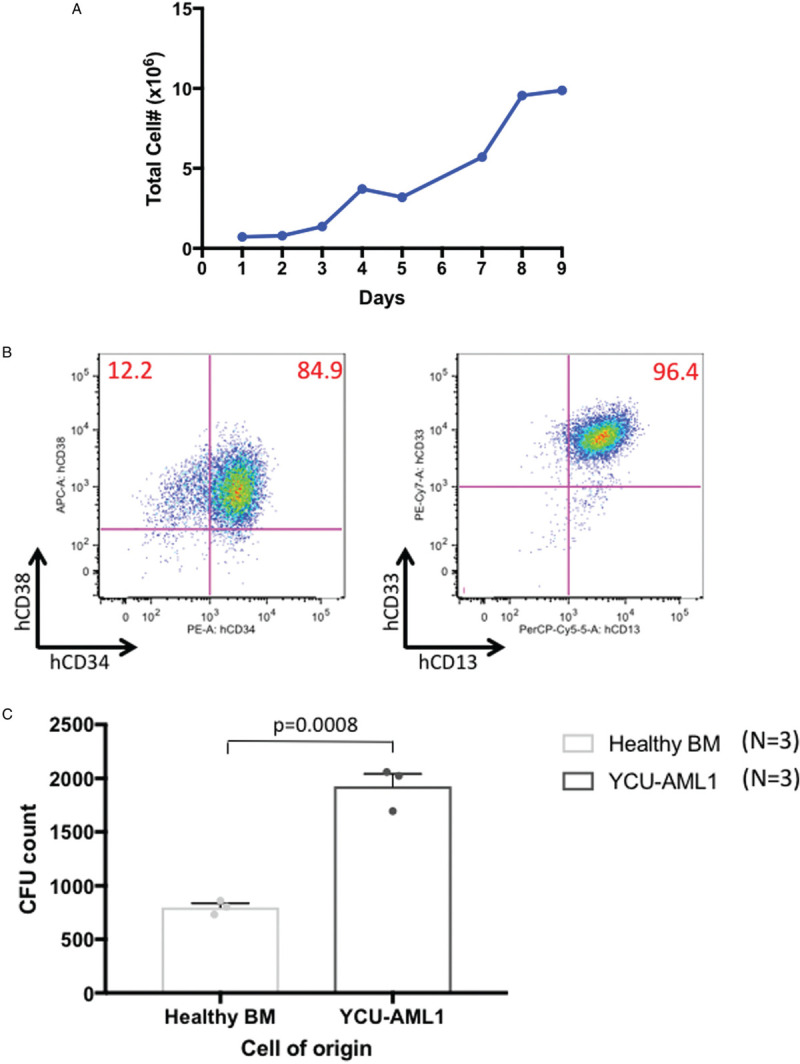
**Characteristics of in vitro cultured YCU-AML1 cells.** A. Growth curve of YCU-AML1 cells in co-culture system with OP9 stromal cells with GM-CSF supplementation. B. Representative immunophenotype of cultured YCU-AML1 cells, with the percentage of each fraction indicated. Cells presented for hCD34/hCD38 and hCD13/hCD33 are gated on hCD45^+^ cells. C. In vitro colony forming assay cultured with human GM-CSF (10 ng/mL) using healthy BMMNCs and YCU-AML1 cells (N = 3 for each group).

Finally, we explored basic characteristics of cultured YCU-AML1 cells in vitro. YCU-AML1 cells proliferated in co-culture system with OP9 stromal cells with GM-CSF supplementation, which resembles the culture condition of OCI-AML20, previously reported AML cell line harboring inv(3) and monosomy 7 (Fig. 8A).^13^ Cultured YCU-AML1 cells maintained high CD34, CD38, CD13 and CD33 positivity as the original case (Fig. 8B). Since YCU-AML1 and OCI-AML20 are both GM-CSF dependent, we asked if leukemia cell with inv(3)/t(3;3) and monosomy 7 are more sensitive to GM-CSF compared to healthy control cells. Intriguingly, YCU-AML1 cells produced higher number of colonies than healthy control cells in response to GM-CSF stimulation, indicating that GM-CSF hypersensitivity is a hallmark of these leukemia cells (Fig. 8C).

### YCU-AML1 PDX model reproduces molecular features of myeloid malignancies with inv(3)/t(3;3)

**Figure 9:**
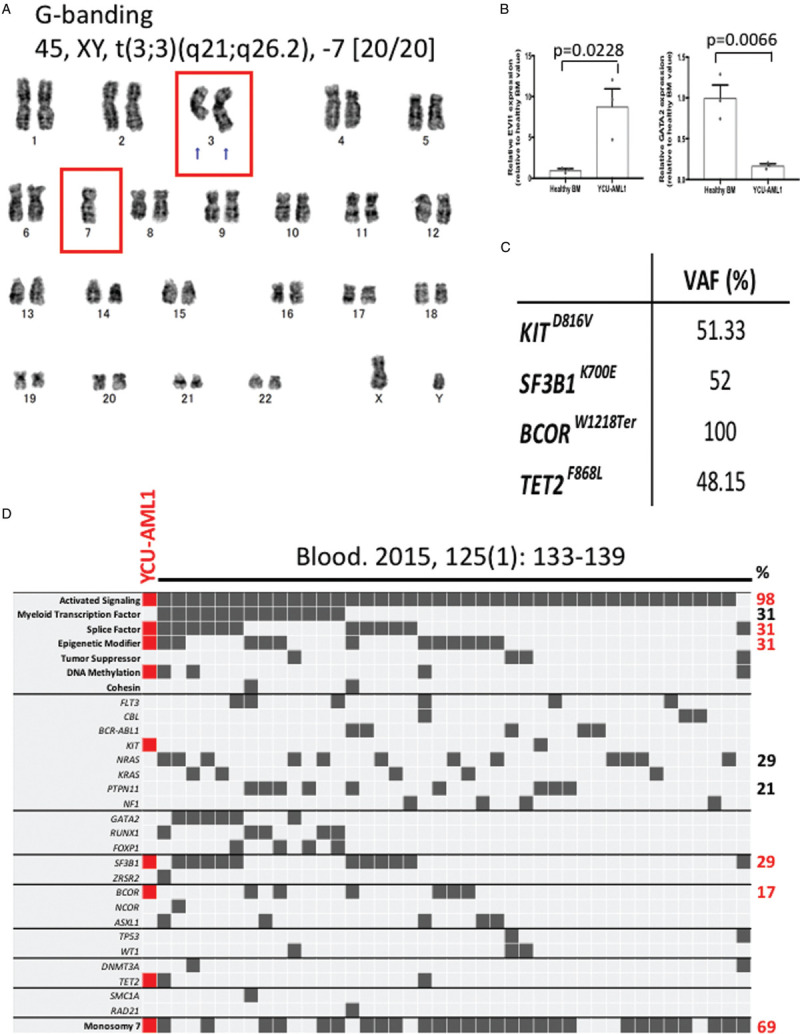
**Molecular features of YCU-AML1 PDX model.** A. An image of G-banding of bone marrow cells derived from primary YCU-AML1 mice. B. RT-PCR for EVI1 and GATA2 using cDNA from CD34^+^ healthy BM and CD34^+^ YCU-AML1 cells (N = 3 for each group). C. Mutations and their variant allele frequencies (VAF) detected in targeted deep sequencing of BM cells derived from primary YCU-AML1 mice. D. Comparison of mutational profile of YCU-AML1 PDX model and those of myeloid malignancies with inv(3)/t(3;3) which are previously published.^14^

In order to unveil molecular basis of leukemic disease in YCU-AML1 mice, we first collected BM cells from moribund mice and performed cytogenetic study. Human chromosome analysis revealed t(3;3)(q21;q26.2) and monosomy 7 in all 20 analyzed cells in metaphases (Fig. 9A). The inv(3) or t(3;3) repositions a distal *GATA2* enhancer to activate *EVI1* expression, and simultaneously confers *GATA2* haploinsufficiency.^17,18^ In line with this, quantitative reverse transcriptase-polymerase chain reaction (RT-PCR) showed higher expression of *EVI1* and lower expression of *GATA2* in CD34^+^ YCU-AML1 cells compared to CD34^+^ healthy control cells (Fig. 9B). Recent studies have uncovered distinct molecular alterations of myeloid malignancies with inv(3)/t(3;3), including predominant mutations in genes involved in RAS/receptor tyrosine kinase (RTK) signaling pathways, splice machinery and epigenetics.^14,19^ Targeted deep sequencing of BMMNCs derived from moribund YCU-AML1 mice revealed heterozygous mutations in *KIT* (*KIT*^*D816V*^, variant allele frequency (VAF) of 51.33%), *SF3B1* (*SF3B1*^*K700E*^, VAF of 52%), *TET2* (*TET2*^*F868L*^, VAF of 48.15%) and an hemizygous mutation in X-linked *BCOR* (*BCOR*^*W1218Ter*^, VAF of 100%) (Fig. 9C). Comparison of these mutational profile with published dataset confirmed that the mutational spectrum of our YCU-AML1 cells fits well with other cases with inv(3)/t(3;3) in terms of acquired mutations in genes involved in 3 recurrent mutational gene categories, such as activated signaling, splice machinery and epigenetics (Fig. 9D). Together, these data suggest that our MDS/AML model precisely recapitulates molecular basis of human myeloid malignancies with inv(3)/t(3;3).^14^

## Discussion

In this study, we established a new high-risk MDS/AML cell line and its PDX model harboring both t(3;3)(q21;q26.2) and monosomy 7, YCU-AML1 cells and YCU-AML1 mice. With regard to the clinical course of the original case, the initial BM exam showed normal karyotype whereas the second BM exam revealed acquisition of t(3;3)(q21;q26.2) and monosomy 7, indicating that these risk-defining cytogenetic abnormalities may have accelerated leukemic transformation. Indeed, AML or MDS with inv(3)(q21q26.2) or t(3;3)(q21;q26.2) defines a high-risk myeloid malignancy with poor prognosis, in which monosomy 7 is the most frequent co-occurring chromosomal abnormality.^3,4^ Although the exact molecular mechanism by which inv(3)/t(3;3) and monosomy 7 preferentially co-occur remains elusive, previous study has shown that overexpression of *EVI1* in human cells disrupts normal centrosome duplication, thereby leading to genomic instability and acquisition of monosomy 7.^20^ Of note, the second BM exam at the time of leukemic transformation exhibited evidence of BM dysplasia in all three hematopoietic lineages including dysmegakaryopoiesis (micromegakaryocytes, nuclear hypolobation, bilobated, multinucleation), a major morphological features in inv(3)/t(3;3) MDS.^4^

The established YCU-AML1 mice demonstrate blast emergence in PB, pancytopenia and marked splenomegaly. Given hepatosplenomegaly is common in human inv(3)/t(3;3) AML, our PDX model likely recapitulates the original disease.^21^ It is noteworthy that YCU-AML1 cells can propagate myeloid leukemia in serial transplant recipients, consistent with the reproducibility of our PDX model. Flow cytometric analysis of the BM and spleen of these mice revealed that two subpopulations (CD34^+^/CD38^+^ and CD34^-^/CD38^+^) were observed in the BM whereas CD34^+^/CD38^+^ fraction was the only major population in the spleen, suggesting functional difference of the BM and spleen as a leukemic niche. Recent study has shown that *Scf* and *Cxcl12* expressed by endothelial cells and *Tcf21*^+^ stromal cells around sinusoids in the red pulp drive extramedullar hematopoiesis in the spleen in response to diverse hematopoietic stress which is distinct from BM niche.^22^ Functional heterogeneity of CD34^+^ hematopoietic stem/progenitor cells is also reported where splenic CD34^+^ cells are enriched for malignant stem cell compartment compared to peripheral CD34^+^ cells in patients with myelofibrosis.^23^ These studies clearly agree with the notion that BM and spleen have a distinct role as a leukemic microenvironment.

YCU-AML1 cells continuously propagated in co-culture system with OP9 stromal cells and GM-CSF supplementation for more than 3 months. This culture condition is analogous to that of OCI-AML20 cells, another AML cell line harboring inv(3) and monosomy 7.^13^ Previous studies have shown that AML cells with high *EVI1* expression exhibit increased expression of *G protein-coupled receptor 56* (*GPR56*) or *Integrin α6* (*ITGA6*), leading to enhanced cellular adhesion ability and drug resistance to cytarabine.^24,25^ On the other hand, AML xenograft efficiency is significantly improved in NSG mice constitutively expressing human stem cell factor (SCF), GM-CSF and interleukin-3 (IL-3),^26^ suggesting that these hematopoietic cytokines can support expansion and engraftment of AML cells in vivo. Thus, both YCU-AML1 and OCI-AML20, two AML cell lines harboring inv(3)/t(3;3) and monosomy 7, share cell adhesive feature of AML cells with high *EVI1* expression with hypersensitivity to GM-CSF.

Targeted deep sequencing of BMMNCs derived from moribund YCU-AML1 mice revealed mutations in *KIT*, *SF3B1*, *TET2* and *BCOR*. Mutational profile of our MDS/AML model is consistent with those of other cases with inv(3)/t(3;3), indicating that our MDS/AML model accurately represents molecular features of human myeloid malignancies with inv(3)/t(3;3).^14,19^ Importantly, mutation in *SF3B1* is frequent in MDS with ring sideroblasts (MDS-RS), being present in 80-90% of MDS-RS with single lineage dysplasia (MDS-RS-SLD) cases and 30–70% of MDS-RS with multilineage dysplasia (MDS-RS-MLD) cases.^27^ Moreover, mutations in *TET2* and *DNMT3A*, genes affecting DNA methylation, are associated with *SF3B1* mutation and are more common in MDS-RS-MLD than in MDS-RS-SLD.^27^ These observations clearly coincide with our original case who showed morphological evidence of multilineage dysplasia with ring sideroblasts harboring both *SF3B1* and *TET2* mutations. Recent studies have uncovered functional cooperativity of oncogenic disease alleles in myeloid transformation. Concurrent loss of *Tet2* and *Bcor* in murine hematopoietic compartment leads to lethal MDS development in vivo.^28^ In addition, simultaneous loss of *Tet2* and expression of *Sf3b1*^*K700E*^ or *cKit*^*D814V*^ in murine hematopoietic cells induces MDS or myeloproliferative neoplasm (MPN) like disease in vivo,^29,30^ suggesting that multiple combinations of leukemic disease alleles found in our MDS/AML model have driven initiation of MDS and leukemic transformation in the original case. Above all, we and others have confirmed that around 98% of MDS/AML cases with inv(3)/t(3;3) have mutations in genes involved in RAS/RTK signaling pathways and around 30% of patients possess *SF3B1* mutation.^14^ These data strongly indicate leukemogenic relevance of combinations between inv(3)/t(3;3) and RAS/RTK signaling or *SF3B1* mutation, which require future mechanistic investigation.

Taken together, we established a new MDS/AML cell line, YCU-AML1, and its PDX model harboring both t(3;3)(q21;q26.2) and monosomy 7, two recurrent high-risk cytogenetic abnormalities seen in AML and MDS. YCU-AML1 cells show characteristic features of a 3q-rearranged AML, such as elevated *EVI1*/reduced *GATA2* expression and cytokine dependency. Clinicopathological findings as well as mutational spectrum of our PDX model highly recapitulate phenotypic/genotypic features of myeloid malignancies with inv(3)/t(3;3). YCU-AML1 will be available to the research community upon request and we believe our model will serve as a valuable tool for future studies exploring pathogenesis of high-risk MDS/AML with t(3;3)(q21;q26.2) and monosomy 7.

## Methods

### Patient

A bone marrow sample of a 62-year-old male patient diagnosed with AML-MRC (MDS/AML) was obtained at Keio University School of Medicine. The patient first presented in June 2011 and the sample from which the PDX model was established was collected in June 2012 when the patient suffered from leukemic transformation. The patient provided informed consent. Approval was obtained from the Institutional Review Board at Keio University School of Medicine and Yokohama City University Graduate School of Medicine, and conducted in accordance with the Declaration of Helsinki protocol.

### Animals

NOD/SCID-IL2Rγ^null^ (NSG) mice were purchased from Charles River Laboratories Japan (Yokohama, Japan). All animal procedures were conducted in accordance with the Guidelines for the Care and Use of Laboratory Animals and were approved by the Institutional Animal Care and Use Committees at Yokohama City University Graduate School of Medicine.

### Histological evaluation and immunohistochemistry

Patient BM trephine biopsy, murine spleen and vertebra specimens are fixed by 10% neutral buffered formalin, ethylenediaminetetraacetic acid (EDTA) decalcified and paraffin embedded. Pathological specimens are stained by conventional hematoxylin and eosin (H&E) staining and immunohistochemistry staining for CD34 and CD38. BM and peripheral blood (PB) smears are stained by ASD-Giemsa staining to evaluated cellularity and myeloid-per-erythroid ratio. To immunostain CD34 (O.Bond/10, 1:1; Leica) and CD38(AT13/5, 1:100; Leica), we used the Leica BOND-MAX (Leica Biosystems, Wetzlar, Germany), automated immunohistochemistry stainer. Histological diagnoses were performed by 3 pathologists (IK, ME, and KK).

### Establishment of YCU-AML1 cells

Cryopreserved BM cells from the xenograft were cultured in co-culture system with OP9 stromal cells in Iscove medium containing 10% fetal bovine serum, 100U/100 μg/mL penicillin/streptomycin (Gibco, MA, USA), 55 μM beta mercaptoethanol (Sigma-Aldrich, MO, USA) and 20ng/mL GM-CSF (PeproTech, NJ, USA). Cells were replaced onto fresh OP-9 stromal cells every week.

### Bone marrow transplantation

To generate primary YCU-AML1 mice for basic and survival analysis, 1 ×  10^6^ BMMNCs derived from the patient were transplanted via tail vein injection into sublethally irradiated (2.4Gy) NSG host mice. To generate secondary YCU-AML1 mice, 1 × 10^6^ cryopreserved BM cells derived from primary YCU-AML1 mice were transplanted via tail vein injection into sublethally irradiated (2.4Gy) NSG host mice. For limiting dilution assay, 1.0 × 10^6^, 1.0 × 10^5^, 1.0 × 10^4^, 1.0 × 10^3^, or 1.0 × 10^2^ cryopreserved BM cells derived from primary YCU-AML1 mice were transplanted via tail vein injection into sublethally irradiated NSG host mice (N = 4 for each group).

### Peripheral blood and bone marrow analysis

Blood was collected by tail cut using EDTA-coated microhematocrit capillary tubes (Vitrex Medical A/S, Herlev, Denmark). Automated peripheral blood counts were obtained using a Celltacα (NIHON KOHDEN, Tokyo, Japan) according to the manufacturer's protocol. Total bone marrow cell number was enumerated by Vi-Cell XR Cell Counter (Beckman Coulter, CA, USA).

### Flow cytometry

For surface flow cytometry of mouse PB, BM, and spleen, red blood cells (RBCs) were lysed and stained with monoclonal antibodies in PBS plus 1% BSA for 1 hour on ice. Cell populations were analyzed using a BD FACS Canto II or BD FACSCelesta Flow Cytometer (Becton Dickinson, NJ, USA). Data were analyzed with FlowJo software (Tree Star, NJ, USA).

### Antibodies

Antibodies used for flow cytometry were as follows: (anti-mouse) CD45.1 (A20), (anti-human) CD45 (2D1), CD34 (561), CD38 (HIT2), CD13 (WM15) and CD33 (WM53). All antibodies were purchased from BioLegend (CA, USA).

### Cell growth assay

YCU-AML1 cells were seeded in 6 well plate with OP9 stromal cells at a density of 0.25 × 10^6^ cells/mL in Iscove medium containing 10% fetal bovine serum, 100 U/100 μg/mL penicillin/streptomycin (Gibco, MA, USA), 55 μM beta mercaptoethanol (Sigma-Aldrich, MO, USA) and 20ng/mL GM-CSF (PeproTech, NJ, USA). Total cell numbers were counted using Vi-Cell XR Cell Counter (Beckman Coulter, CA, USA).

### In vitro colony forming assays

For cytokine sensitivity assay, healthy BMMNCs or YCU-AML1 cells were seeded in 6 well plate at 2.0 × 10^5^ cells per well density in triplicate into cytokine free methylcellulose medium (Methocult H4230, STEMCELL Technologies, Vancouver, Canada) with GM-CSF 10 ng/mL. Plates were placed into an incubator at 37°C and 5% CO_2_ for 14 days and colony counts were determined. Healthy human BMMNCs were purchased from STEMCELL Technologies (Vancouver, Canada).

### Cytogenetic studies

Whole bone marrow cells were collected from primary YCU-AML1 mice and were cultured in RPMI medium supplemented with 10% fetal bovine serum. Cell cycle arrest was induced at cell growth phase by adding KaryoMAX Colcemid solution (Gibco, MA, USA). After hypotonic treatment with potassium chloride, cells were fixed with Carnoy Solution (Fuji Film/Wako Junyaku, Osaka, Japan) and mounted on a slide glass. Chromosomes were banded by trypsin-Giemsa. Karyotypes were described according to the guidelines of the International System for Human Cytogenetic Nomenclature.

### Isolation of CD34^+^ cells

Isolation of CD34^+^ cells was performed with EasySep Human CD34 Positive Selection Kit II (17856, STEMCELL Technologies, Vancouver, Canada) and EasySep Magnet (18000, STEMCELL Technologies, Vancouver, Canada).

### Quantitative RT-PCR

Total RNA was isolated from CD34^+^ cells of healthy human BM or YCU-AML1 cells using RNeasy Mini Kit (Qiagen, Venlo, Netherland) according to the manufacturer's protocol. RNA was treated with RNase-free DNase Set (Qiagen, Venlo, Netherland) to remove contaminating genomic DNA. cDNA was synthesized using ReverTra Ace qPCR RT Master Mix (TOYOBO, Osaka, Japan). The quantity of cDNA was normalized according to the expression of human *HPRT* measured by real-time RT-PCR using THUNDERBIRD SYBR qPCR Mix (TOYOBO, Osaka, Japan) and StepOnePlus Real-Time PCR System (Applied Biosystems, CA, USA). Data were analyzed by the delta Ct ratio technique using housekeeping genes. The sequences of the primers used for the amplification of each gene are as follows: human *EVI1* (Forward primer 5’-TGAGGATGACTATGAAGAAACCAGT-3’, Reverse primer 5’-GCAGAAAGTCCACTTTTATATTCTTCC-3’), human *GATA2* (Forward primer 5’-ACTCCTTCACTCTCAGAGGC-3’, Reverse primer 5’-AGAAGACGTCCACCTCGTCT-3’), human *HPRT* (Forward primer 5’-GGACAGGACTGAACGTCTTGC-3’, Reverse primer 5’-CTTGAGCACACAGAGGGCTACA-3’). Healthy human CD34^+^ BM cells were purchased from STEMCELL Technologies (Vancouver, Canada).

### Targeted deep sequencing of myeloid panel genes

Whole BM cells were collected from bilateral femurs, tibias and iliac bones of primary YCU-AML1 mice and total DNA and RNA were isolated using AllPrep DNA/RNA Mini Kit (Qiagen, Venlo, Netherland). The Oncomine Myeloid Research (OMR) panel (Thermo Fisher Scientific, MA, USA) consists of RNA- and DNA-based gene panels with 526 DNA and 700 RNA amplicons. The panel targets the complete exonic regions of 17 genes, exonic hot spots of 23 genes, 29 fusion genes, 5 expression genes, and 5 control genes.^15^ OMR panel library preparation was performed by manual and automated processes, according to the manufacturer's protocols (Thermo Fisher Scientific, MA, USA). Briefly, barcoded libraries were generated from 10 ng of sample genomic DNA and RNA. Targets were amplified using an RNA primer pool and 2 DNA primer pools using highly multiplexed PCR amplification. The amplicons were partially digested with Fupa reagent, followed by ligation of unique barcode adapters for each library. The barcoded libraries were normalized to 100pmol/L using the Ion Library Equalizer Kit (Thermo Fisher Scientific, MA, USA). Automated library preparation was performed using the OMR Assay-Chef Ready kit on the Ion OneTouch 2 System (Thermo Fisher Scientific, MA, USA). The normalized DNA and RNA libraries were diluted to optimized concentration and combined at a ratio of 80:20. Templating was performed on the Ion OneTouch 2 System, according to manufacturer's protocols. The sample library was clonally amplified onto Ion Sphere Particles by emulsion PCR with the Ion OneTouch 2 System in line with the manufacturer's protocols. Enriched Ion Sphere Particles were loaded onto 530 chips using an Ion 520 and Ion 530 Kit. Sequencing was performed on an Ion PGM Sequencer (Thermo Fisher Scientific, MA, USA). Sequence alignment to reference genome hg19 and base calling were performed using the Torrent Suite software version 5.10.0 (Thermo Fisher Scientific, MA, USA). Variant identification and annotation were performed using Ion Reporter (IR) software version 5.10 (Thermo Fisher Scientific, MA, USA). Coverage maps were generated using the Coverage Analysis plugin version 5.10.0 (Thermo Fisher Scientific, MA, USA). The Ion Reporter default analysis parameter settings for Oncomine Myeloid Research workflow were used. In these settings, the minimum coverage requirement for the analysis is 20 for both SNVs and indels and 15 for hot spots; the minimum cutoff variant allele fraction (VAF) is 2.5% for both SNVs and indels and 3% for hot spots; and the maximum strand bias tolerance is 0.9 for SNVs, 0.85 for indels, and 0.96 for hot spots. A separate custom DNA Myeloid filter chain was generated in IR that allows for all possible pathogenic variants at a VAF≤ 1%. The custom filter chain settings were as follows: variant type: SNV, indel, MNV, CNV, longdel, fusion, ExPR_Control, Gene_Expression, RNAXonVariant, ProcControl, and *FLT3*-ITD; variant effect: effect in missense, non-frame shift Insertion, non-frame shift Deletion, non-frame shift Block Substitution, nonsense, stoploss, frame shift Insertion, frame shift Deletion, and frame shift Block Substitution; 0 ≤ VAF ≤ 1%. The default RNA filter chain, oncomine variant, was used.

### Statistical analysis

The unpaired Student's *t* test was used to compare the mean of two groups. p values < 0.05 were considered statistically significant. Data were analyzed and plotted using GraphPad Prism 7 software (CA, USA). Data shown in graphs indicate mean ± S.E.M. LSC frequency was calculated by limiting dilution assay and regression analysis using L-Calc software (STEMCELL Technologies, Vancouver, Canada). Kaplan-Meier survival analysis and log-rank test were used to compare survival outcome.
